# The complete chloroplast genome of *Medicago ruthenica* cv. ‘Taihang’ (Fabaceae) from Shanxi, China

**DOI:** 10.1080/23802359.2021.1966328

**Published:** 2021-08-18

**Authors:** Hong-yu Xu, Yu-ying Li, Xiao-lan Shang, Hua Zhong, Kuan-hu Dong, Fang-shan Xia

**Affiliations:** College of Grassland Science, Shanxi Agricultural University, Jinzhong, Shanxi, China

**Keywords:** Chloroplast genome, *Medicago ruthenica*, phylogenetic analysis

## Abstract

*Medicago ruthenica* is an important perennial forage with multiple characteristics of resistance. In this study, we sequenced and characterized the complete chloroplast genome of *M. ruthenica* ‘Taihang’, which is 124, 254 bp in length. A total of 108 genes were identified, including 74 protein-coding, 30 tRNA, and four rRNA genes. Phylogenetic analysis based on 27 chloroplast genomes showed that *M. ruthenica* ‘Taihang’ has a close relationship with *M. ruthenica* from Qinghai Province, China. The data are useful in better understanding the genetic diversity and stress resistance of *Medicago* and contribute to the phylogenetic study of Trifolieae.

*Medicago* L. is classified in the Fabaceae and is an important forage plant because of its rich nutrition and digestibility (Choi et al. 2019). *Medicago ruthenica*, with the characteristics of cold resistance, drought resistance, salt and alkali resistance, infertility resistance (Shu et al. 2018), is equipped with wide adaptability which makes the morphology vary greatly under different environmental conditions (Cui et al. 1998). In China, three cultivars have been described as cultivars of *M. ruthenica*, including ‘Tumote’, ‘Zhi Lixing’ and ‘Taihang’. The cultivar ‘Taihang’, which has a large number of leaves, good quality, high grass yield and seed yield, is more suitable for planting in northern semi-arid areas in China and other similar conditions. To date, several barcode analyses have been performed using *matK* and *trnH-psbA* (Nadia et al. 2014; Badr et al. 2020), and a complete chloroplast genome of *M. ruthenica* from Qinghai Province, China was assembled and published previously (Xie et al. 2021). In this research, we aimed to establish and characterized the chloroplast (cp) genome of the ‘Taihang’ cultivar to provide additional genomic data for the phylogenetic study of this species and the Trifolieae.

In this study, fresh leaves of *M. ruthenica* ‘Taihang’ were collected from experimental plots of the Shanxi Agricultural University of China, located at 112.5824 E, 37.4183 N. The voucher specimen is stored in the herbarium of Shanxi Agricultural University (Specimen No. SXAU-CoGS-20Mr01, Hua Zhong, zhonghua109@126.com). Chloroplasts were isolated from 5 g of fresh leaves by gradient centrifugation on Percoll. Then, the chloroplast DNA was extracted using the CTAB method (Doyle JJ and Doyle JL 1987). A 350 bp DNA library was constructed and PE-150 bp reads were sequenced on an Illumina Hiseq 2500 platform. To filter reads from the chloroplast genome, approximately 348.4 Mb of trimmed high-quality reads were mapped to the previously published cp genomes (NC_042841.1, NC_042849.1, NC_042854.1, NC_003119.8 and NC_032066.1) Minimap2 v2.13-r850 with default parameters (Li 2018). The resulting aligned reads were co-assembled using GetOrganelle v1.7.3.1 (Jin et al. 2020), and one circular contig was produced. Genome annotation was conducted using the online tool GeSeq (https://chlorobox.mpimp-golm.mpg.de/geseq.html, Tillich et al. 2017) and manually corrected.

The complete chloroplast genome of *M. ruthenica* ‘Taihang’ (GenBank accession number: MW703984.1) has a circular chromosome that is 124,254 bp in length with a GC content of 34.29%. A total of 108 genes were annotated, including 74 protein-coding, 30 tRNA, and four rRNA genes. Fifteen of the genes contain one intron and one of them contains two introns. Compared with the chloroplast genomes previously published about the other plants of *Medicago* L. (Choi et al. 2019; Zhao et al. 2021), *M. ruthenica* (MH901635.1) published by Xie et al. (2021) lacks the gene *ndhD* which encodes NADH dehydrogenase subunit 4, and *M. ruthenica* ‘Taihang’ (MW703984.1) lacks the gene *petN* which encodes cytochrome b6/f complex subunit VIII. In addition, the gene sequence from *clpP* to *psaA* is reversed in *M. ruthenica* MH901635.1 and MW703984.1. The genes *trnC-GCA* and *rpoB* in MH901635.1 are located between *rpoC1* and *psaB*, while they are located between *psbM* and *rpoC1* in MW703984.1 and which is similar to the other plants of *Medicago* L. (Choi et al. 2019). However, the chloroplast genome of two cultivars still shows a high level of gene synteny.

To reveal the phylogenetic position of *M. ruthenica* ‘Taihang’, a maximum-likelihood (ML) tree was constructed using 27 complete chloroplast genome sequences, including 22 *Medicago* species, three *Trifolium* species, and *Melilotus albus* and *Trigonella foenum-graecum* serving as outgroup taxa. All sequences were downloaded from GenBank and were aligned using MAFFT v7.311 using the default settings (Katoh and Standley 2013). The ML tree was produced by MEGA7 v7.0.26 (Kumar et al. 2016) using 1,000 bootstrap replicates and the Tamura-Nei model + Uniform rates nucleotide substitution model. The phylogenetic tree showed that *M. ruthenica* ‘Taihang’ was fully resolved on a branch with *M. ruthenica* (MH901635.1) within a monophyletic clade with other species classified to the genus *Medicago* (Figure 1). In addition, barcode analyses had also been carried out using genes *matK* and *trnH-psbA*, and which presented the same relationship between ‘Taihang’ and other *Medicago* L. plants. The complete analysis of the chloroplast genome of *M. ruthenica* ‘Taihang’ is useful for studying the genetic diversity and stress resistance of *Medicago* plants.

**Figure 1.**
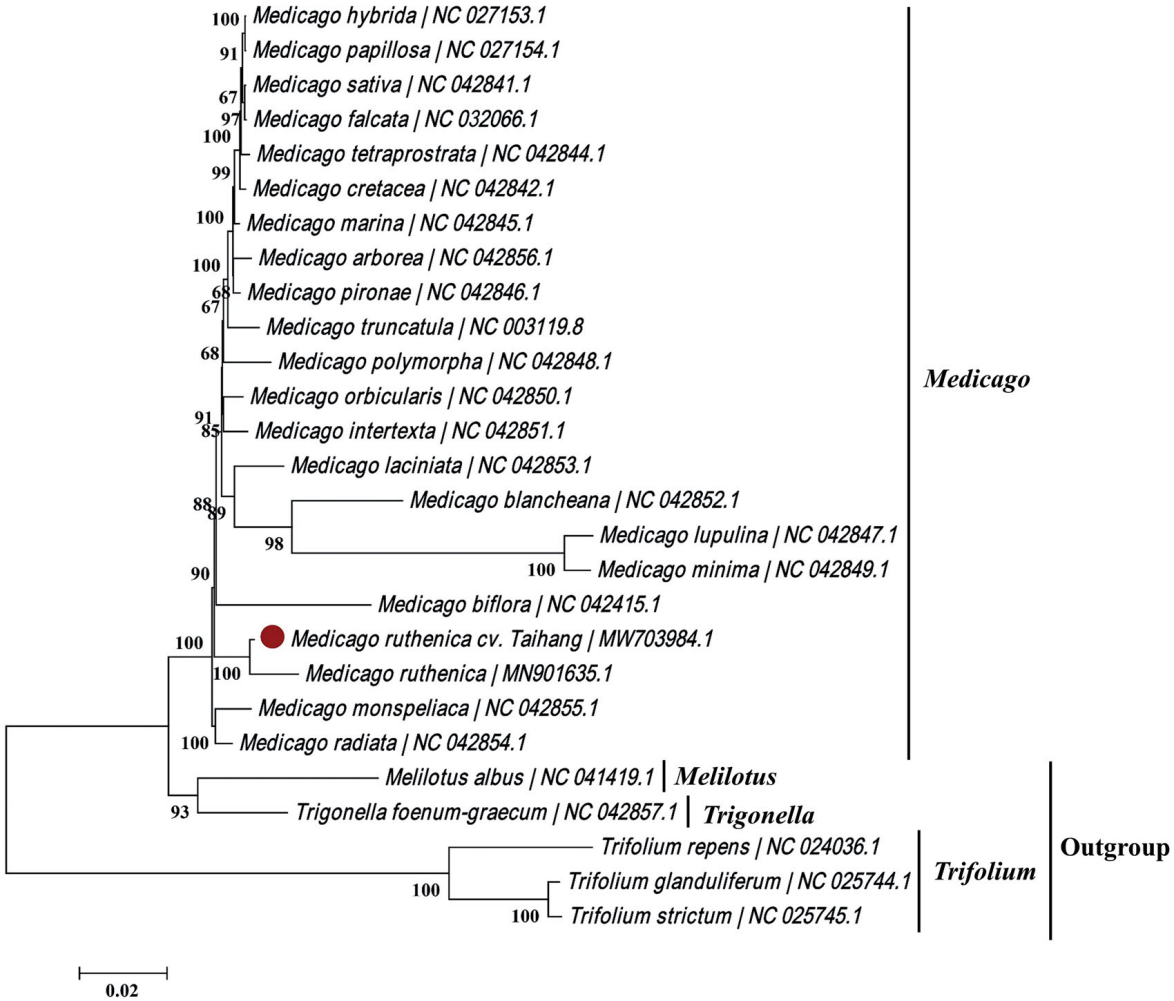
Phylogenetic analysis of 27 species from Trifolieae based on complete chloroplast genome sequences using MEGA7. Bootstrap percentages are based on 1,000 replicates.

## Data Availability

The raw data that support the findings of this study is openly available in NCBI Sequence Read Archive at [https://www.ncbi.nlm.nih.gov/sra/SRR14114639] under the BioProject ID PRJNA718640 and the annotated chloroplast genome has been deposited in Genbank [https://www.ncbi.nlm.nih.gov/nuccore/MW703984.1/] under the reference number MW703984.1.
